# Severe combined immunodeficiency syndrome with RAG1 mutation gene - case report

**DOI:** 10.1186/1939-4551-8-S1-A178

**Published:** 2015-04-08

**Authors:** Ana Lúcia Lima, Hevila Andrade, Erika Sundin, Patricia Loureiro, Juliana Gonçalves, Lúcia Guirau, Aline Umeta, Vanessa Pereira, Andrea Cohon

**Affiliations:** 1 Hospital Infantil Darcy Vargas, Brazil

## Background

Reporting a case of Severe combined immunodeficiency syndrome with RAG1 mutation gene.

## Methods

Analysis of medical records was conducted to obtain detailed clinical history.

## Results

ACOA, 3 months, female, born cesarean with 38 weeks. She had the following vaccines: BCG, hepatitis B, VIP / VOP, tetravalent rotavirus, pneumococcus and menigococo. The patient had daily fever for 4 days, oliguria, dyspnea, and diarrhea with severe septic shock and respiratory failure. The patient remained hospitalized in intensive care for 50 days with tracheal intubation for 23 days. When she was 2 months old was hospitalized for 10 days with septic shock. Requested tests: CBC showing lymphopenia ( 855), positive Rotavirus, hipogamoglubulinemia ( IgG 143, IgM 9.7, IgA 26), absence of thymus chest radiography and immunophenotyping with amendment (Lymphocyte T - CD45/CD3 = 60 cells/mm3, CD45/CD3/CD4 = 55celulas/mm3, CD45/CD3/CDCD8 = 5 cells/mm3, CD4/CD8 ratio = 11.00, B lymphocyte - CD45/CD19 celula/mm3 = 1, NK-cells - CD45 / CD3-/CD16 + / CD56 + = 89 cells/mm3).The results of these tests made the diagnosis of severe combined immunodeficiency syndrome (T-B-NK +)(SCID) associated with homozygous mutation in the RAG-1 gene by sequencing of SCID panel ,therapy was initiated with cefepime trimethoprim-sulfamethoxazole, rifampin, isoniazid, ethambutol, pyridoxine, fluconazole,zinc e PRBC. Initiate treatment with human gamma globulin 400mg/kg intravenously for 5 consecutive days and after this repeated doses with intervals 21/21 days. The evolution was favorable ,indicating bone marrow transplantation.

## Conclusions

The Severe Combined Immunodeficiency is a pediatric emergency and it is necessary to increase the clinical suspicion due to a quickly evolution to the death when the treatment is not quickly started. The early diagnosis of the patient in this case results in a better prognosis.

## Consent

Written informed consent was obtained from the patient for publication of this abstract and any accompanying images. A copy of the written consent is available for review by the Editor of this journal.

